# Biosensors Paving the Way to Understanding the Interaction between Cadmium and the Estrogen Receptor Alpha

**DOI:** 10.1371/journal.pone.0023048

**Published:** 2011-08-02

**Authors:** Peter Fechner, Pauliina Damdimopoulou, Günter Gauglitz

**Affiliations:** 1 Institute of Physical and Theoretical Chemistry (IPTC), Eberhard-Karls-University of Tübingen, Tübingen, Germany; 2 Department of Biosciences and Nutrition, Karolinska Institutet, Huddinge, Sweden; University of Akron, United States of America

## Abstract

Cadmium is a toxic heavy metal ubiquitously present in the environment and subsequently in the human diet. Cadmium has been proposed to disrupt the endocrine system, targeting in particular the estrogen signaling pathway already at environmentally relevant concentrations. Thus far, the reports on the binding affinity of cadmium towards human estrogen receptor alpha (hERα) have been contradicting, as have been the reports on the *in vivo* estrogenicity of cadmium. Hence, the mode of interaction between cadmium and the receptor remains unclear. Here, we investigated the interaction between cadmium and hERα on a molecular level by applying a novel, label-free biosensor technique based on reflectometric interference spectroscopy (RIfS). We studied the binding of cadmium to hERα, and the conformation of the receptor following cadmium treatment. Our data reveals that cadmium interacts with the ligand binding domain (LBD) of the ERα and affects the conformation of the receptor. However, the binding event, as well as the induced conformation change, greatly depends on the accessibility of the cysteine tails in the LBD. As the LBD cysteine residues have been reported as targets of post-translational modifications *in vivo*, we present a hypothesis according to which different cellular pools of ERα respond to cadmium differently. Our proposed theory could help to explain some of the previously contradicting results regarding estrogen-like activity of cadmium.

## Introduction

Cadmium is a heavy metal with no known beneficial physiological function. It is ubiquitously present in Earth's crust, from where it is released by volcanic activity, mining, and use of phosphate fertilizers and fossil fuels. Plants take up cadmium from the soil and form the major source of cadmium intake in non-smoking, non-occupationally exposed populations [1]. The average daily intake of cadmium through diet is approximately 10–20 µg, which is close to the recently revised tolerable weekly intake of 2.5 µg/kg set by the European Food Safety Authority [1]. Chronic exposure to cadmium causes damage to the kidneys and bone, increases the risk of various cancers, and disrupts reproductive functions in both female and male [2], [3], [4]. The Interactional Agency for Research on Cancer has classified cadmium as a group I human carcinogen [5]. More recently, studies demonstrating estrogen-like activity of cadmium have raised concerns and led to a classification of cadmium as an endocrine disrupter [6], [7], [8].

The estrogen receptors ERα and ERβ are nuclear hormone receptors that regulate gene expression in response to the female sex steroids estrogens. When the cognate ligand 17β-estradiol (E_2_) binds to the ligand binding pocket within the ERαLBD, it interacts with specific amino acid residues (glu353, arg394, and his524) leading to a conformational change and the formation of the activation function 2 (AF-2), which is an interaction site for co-activators [9]. The recruitment of co-activators bridges the receptor to the basal transcription machinery and allows the regulation of transcription. Alternatively, the activation of ERα can lead to rapid, extranuclear and thus non-genomic effects like release of secondary messengers and activation of kinases [10]. The mechanisms of the extranuclear activities of the ERs remain less well understood than the genomic activities. Nevertheless, inappropriate regulation of ER activity by environmental endocrine disrupters is believed to be a factor behind the increasing incidence of hormonal cancer in industrialized countries. For example, cadmium exposure is linked to the risk of endometrial and breast cancer in humans [11], [12]. These epidemiological connections to increased risk of hormonal cancers in humans render the understanding of the endocrine disruptive mechanisms of cadmium very important.

In mammalian cell culture, cadmium induces the expression of estrogen target genes, triggers activation of cytoplasmic kinases, and promotes proliferation of estrogen responsive cell lines [7], [8], [13], [14], [15], [16], [17], [18], [19], suggesting that cadmium promotes an agonist conformation of the ER and activates both genomic and non-genomic estrogen signaling. However, these effects are not observed in all studies [19], [20], [21], [22]. Similarly, in rodents cadmium promotes uterine growth, the hallmark of estrogen exposure, in some [6], [23], [24] but not all studies [23], [25], [26]. The binding of cadmium to hERα has been studied in two independent reports. Stoica *et al.* reported cadmium as a strong ligand for the hERα (dissociation constant K_D_ = 5*10^−10^ M) [8], while Rider *et al.* classified cadmium as a non-binder [8], [27].

**Figure 1:**
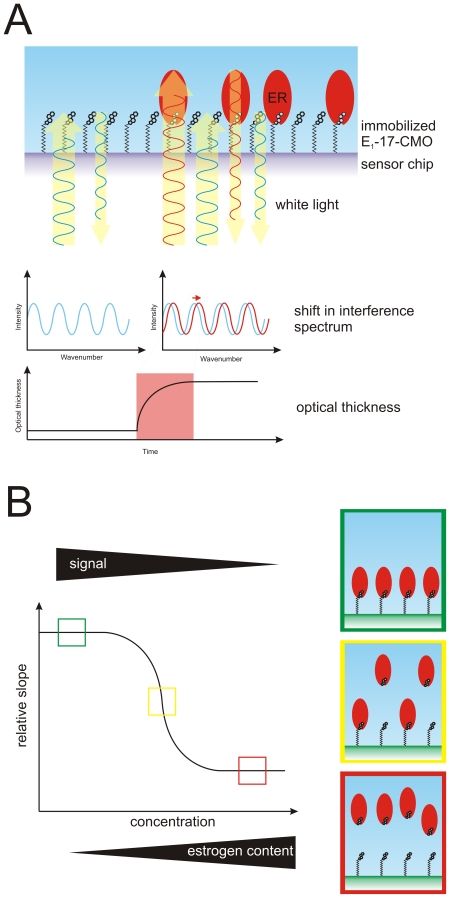
The principle of the RIfS ligand binding assay. (A) The RIfS ligand binding assay relies on derivatives of the specific ERα ligand E_1_-17-CMO that are covalently bound to the sensor chip surface. White light is illuminated into the system, and it reflects from the different boundaries of the multilayered chip. In case protein binds to the surface, the reflectivity is changed and the optical thickness, determined from the interference spectra, increases. (B) The dependence of the optical thickness of estrogen content in the sample. In case there are no ERα ligands present in the sample, the receptor is free to bind the surface, and the optical thickness of the surface is high (the situation boxed with green). If there are ERα ligands present, they prevent the receptor from binding the surface leading to decreased optical thickness (the situation boxed with yellow), until all receptor is saturated with the ligand and it can no longer bind the surface (the situation boxed with red).

In order to help explain these contradictory findings, we decided to apply a novel biosensor technique to study the binding of cadmium to hERα and the conformational consequences of the interaction. Our methodology is based on RIfS, and it represents a label free, time-resolved method for the study of specific interactions between biomolecules. The platform has successfully been applied to ERα before to study the binding to ligands [28], [29], DNA [30], and co-activators [31]. The principle of the RIfS assay relies on measuring the change in optical thickness of thin transducer chips that are coated with suitable biomolecules (Figure 1). We applied two different chips in the current study that we have recently thoroughly characterized: one coated with a derivative of the ERα ligand estrone (E_1_) [29] and another with a peptide that binds to the agonist conformation of ERαLBD [31].

## Materials and Methods

### Materials

RIfS-transducer chips of 1 mm thick D263 glass-substrate with a first layer of 10 nm Ta_2_O_5_ and a layer of 330 nm SiO_2_ on top were obtained from Schott AG (Mainz, Germany). Common organic compounds and biochemicals were purchased either from Fluka (Neu-Ulm, Germany), SigmaAldrich (Deisenhofen, Germany) or Merck (Darmstadt, Germany). 3-Glycidyloxypropyl-trimethoxysilane and diisopropylcarbodiimide were purchased from Fluka, di-amino-poly(ethylene glycol) (DAPEG) with a molecular mass 2000 Da from Rapp Polymere (Tübingen, Germany), and d-biotin and 2-(1H-benzotriazol-1-yl)-1,1,3,3-tetramethyluronium tetrafluoroborate from Sigma-Aldrich. Estrone-17-N-carboxymethyloxime (E_1_-17-CMO) was kindly provided by Dr. Ram Abuknesha (King's College London, UK).

Purified (single band on native-PAGE) carboxymethylated hERαLBD (CM-hERαLBD) (30 kDa) was kindly provided by KaroBio AB (Stockholm, Sweden). Biotinylated peptide α/β I with the amino acid sequence Ser-Ser-Asn-His-Gln-Ser-Ser-Arg-Leu-Ile-Glu-Leu-Leu-Ser-Arg was purchased from Thermo Scientific (Ulm, Germany) and the recombinant full length hERα (66.4 kDa) from Mobitec (Göttingen, Germany).

### Preparation of the transducer chips

The RIfS transducer chips were cleaned, activated and silanized, and diamino-poly(ethylene glycol) was then immobilized to the surface as described previously [29], [31].

The chips coated with E_1_-17-CMO were prepared essentially as described in [29]. Shortly, E_1_-17-CMO was dissolved in dimethylsulfoxide (DMF), N,N-diisopropylethylamine was added, and the mixture was pipetted onto a DAPEG transducer and sandwiched with another DAPEG transducer. The sandwich was then incubated in a DMF-saturated atmosphere for 6 h and finally rinsed with DMF and water.

The chips coated with α/β I peptide were prepared like described before [31]. Shortly, biotin was immobilized to the surface via 2-(1H-benzotriazol-1-yl)-1,1,3,3-tetramethyluronium tetrafluoroborate activation. Then streptavidin solution was flushed over the sensor surface, followed by biotinylated α/β I peptide solution.

A detailed reaction scheme of the surface chemistry is provided in Figure S1.

### Ligands and reaction mixtures

Stock solutions of 1 mg/mL of E_2_ and 4-hydroxy-tamoxifen (4-OHT) were prepared in dimethylsulfoxide and stored at 4°C in the dark until use. CdCl_2_ was stored as 0.1 M stocks in the freezer. Different concentrations of CdCl_2_ were freshly prepared by dilution with Milli-Q water.

The reactions between ERα and ligands were carried out in a binding inhibition test format, where the receptor is pre-incubated with the ligands before the mixture is injected to the RIfS setup. All incubations were carried out for 60 min at 4°C in the dark.

#### Ligand binding assay

In ligand binding assays, a constant concentration of the receptor (548 nM for hERα and 208.3 nM for CM-hERαLBD) was incubated with different concentrations of CdCl_2_ (2.9 nM–285.7 µM). These mixtures were then investigated via RIfS using E_1_17-CMO modified transducers. When E_2_ was titrated to CM-hERαLBD with or without 5 µM CdCl_2_ the concentration of E_2_ was varied between 3.3 nM E_2_ and 1.3 µM E_2_.

A detailed scheme for the binding assay is provided in Figure S2.

#### Conformation assay

In the conformation assay, constant concentrations of receptors (3.3 µM) were incubated with 3.3 mM CdCl_2_, 9 mg/L E_2_ (33 µM), or 9 mg/L 4-OHT (24 µM). These mixtures were then investigated via RIfS using α/β I modified transducers.

A detailed scheme for the conformation assay is provided in Figure S3.

### The principle of the RIfS-based ligand binding assay

The RIfS setup consists of a halogen white-light source and a Y-optical fiber, which guides the light to the transducer chip. The reflected light is travels through the same optical Y-fiber to a diode array spectrometer (Spekol-1100, Analytik Jena, Germany). The liquid handling system consists of a Hamilton dilutor Microlab (Hamilton, Switzerland) with two syringe pumps and a 4-way valve. Data acquisition and evaluation was performed using internal software.

The binding of the receptor to the surface was monitored for 250 s after a 100 s baseline period, followed by a 300–600 s dissociation phase and a regeneration step with 6 M guanidiniumhydrochloride pH 2 and a 240 s baseline period. The optical thickness was followed by recording interference of white light reflected at the interfaces of the chip by a diode array spectrometer. Binding curves were recorded as changes of the apparent optical thickness [nm] versus time [s]. All measurements were carried out in 500 mM Tris buffer containing 100 mM KCl with a pH of 7.4 at room temperature (∼25°C). The assays were repeated three times and in figures mean optical thickness with standard deviation is presented.

The concentration of the receptor in the reaction mixture was adjusted so that the binding signals revealed a linear shape. By providing an excess of possible interaction partners on the surface, it could be ensured that every free receptor molecule could bind to a superior number of free immobilized ligands on the surface. Under these conditions, the obtained sensor signal is dependent on the diffusion of receptors to the sensor surface expressed by Fick's law,
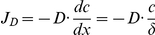
where 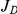 is the diffusion flux, 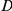 the diffusion coefficient of the biomolecules in solution, 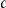 the concentration of these biomolecules, and 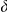 the thickness of the diffusion layer. The slope of the binding curve is proportional to the concentration of free receptor in solution. Assuming that Cd binds to the same binding pocket as the steroidal estrogens, such as E_2_, the equation for the affinity constant deducted from the mass equation results in the following formula,
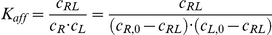
where 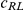 is the concentration of the receptor-ligand complex, 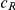 the concentration of receptor, 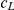 the concentration of ligand, 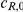 the initial concentration of receptor, and 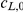 the initial concentration of ligand. The concentration of receptor-ligand complex is accordingly
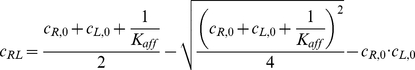



The concentration of free receptor can therefore be expressed as
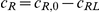



Knowing this relation between the equations it is possible to determine the affinity constant directly as a parameter of the fit function.

## Results and Discussion

### Measurement of biomolecular interactions using RIfS

Biosensors represent relatively young analytical methodology, but have proven to be very powerful in combination with nuclear receptors [32]. Among the large group of biosensors, the label-free techniques provide particular advantages. Their ability to trace biomolecular interactions in a time-resolved manner without a need for labeling minimizes the possibility of artifacts. In addition, in contrast to other more popular biosensors, such as surface plasmon resonance, RIfS uses glass instead of metal films as transducers. In other words, the RIfS surface chemistry does not rely on thiol/gold chemistry which is a great advantage in the present study considering the possible interaction between thiol-containing polymers and cations such as cadmium.

We applied two different sensor chips in our studies, one coated with the ER specific ligand E_1_-17-CMO, and one coated with a peptide called α/β I that binds to the AF-2, *i.e.* the agonist conformation of ERαLBD. Both assay formats have been developed and characterized by us [29], [31]. The principle of the RIfS based binding assay is presented in Figure 1 (and discussed in detail *e.g.* in [33]). We first pre-incubated the receptor protein in solution with CdCl_2_ or control ligands, and then introduced the mixture to the sensor chips. The binding of the receptor to the surface was monitored over time with the help of white light passing through the chip, and the reflected light was recorded with a diode array spectrometer. Association (and dissociation) of the receptor to the surface changes the reflectivity of the multilayered chip, which is seen as changes in the interference spectrum, and can be calculated as optical thickness of the chip (Figure 1A). Using the curve showing the change in optical thickness over time we then calculate the relative slope, which is simply defined as the change of optical thickness over time in a given treatment. Finally we normalize the results in one series of experiments to the highest observed slope, which is normally seen in the blank sample containing untreated receptor. For example, in case cadmium does not bind to ERα ligand binding pocket, the receptor is free to bind to the E_1_-17-CMO coated surface, which we observe as an increase in the optical thickness, which gives a high relative slope (Figure 1B). Similarly, in the conformation assay, if cadmium induces an agonist conformation, the receptor will bind to the α/β I coated surface, and the optical thickness is increased.

### Binding of cadmium to hERα

**Figure 2:**
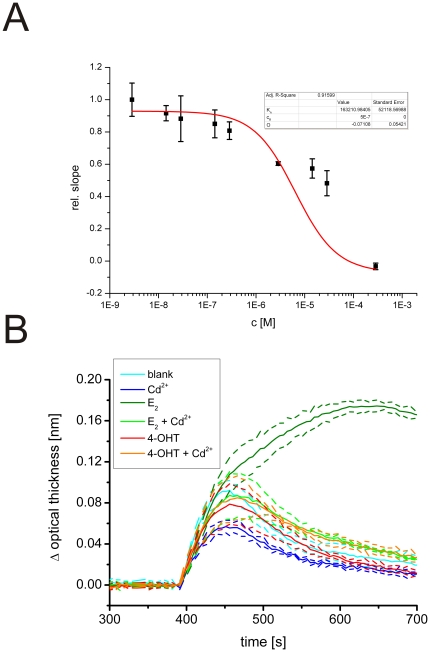
Effect of cadmium on hERα. (A) The binding of cadmium to hERα was studied by incubating different concentrations of CdCl_2_ (2.9 nM–285.7 µM) with a constant concentration (c_R_ 548 nM) of hERα. The mixtures were then guided to the sensor surface and the optical thickness monitored. Cadmium dose dependently reduces the thickness, indicating that it binds to hERα. The affinity constant (K_A_) is given as a fit-parameter while O is an offset allowing the fit to be adjusted in y-axis direction. (B) The conformation of hERα was assessed after the receptor was incubated with vehicle, CdCl_2_, E_2_, 4-OHT, CdCl_2_+E_2_, or CdCl_2_+4-OHT. The average binding curves (recorded for 700 sec) of three independent assays is shown with the standard deviation depicted as dotted lines. Only E_2_ alone triggered an agonist conformation recognized by the surface. Cadmium alone and in combination with other ligands prevented the formation of agonist conformation.

Currently, there is little doubt that cadmium has an effect on the estrogen signaling. However, it is striking that the nature of the reported effects varies from strong agonist to antagonist in the different model systems [6], [8], [21], [22], [26], [27]. To understand these inconsistencies, it is essential to know how exactly cadmium interacts with the ER. To our knowledge, only two studies have assessed the binding of cadmium to hERα: Stoica *et al.* classified cadmium as a strong agonist while Rider et al. concluded that cadmium is a non-binder that, however, disrupts the binding properties of E_2_ [8], [27]. In both reports, cell based and cell free radioligand binding assays were utilized side by side. To gain new insights into the binding mechanism, we applied the RIfS methodology developed in our laboratory. We incubated different concentrations of CdCl_2_ with a constant concentration of recombinant full length hERα for 1 h, after which we quantified the relative amount of free ERα with the E_1_-coated RIfS sensor chips. Our results show that CdCl_2_ dose-dependently decreases the optical thickness of the surface (Figure 2A). In other words, cadmium binds to hERα. In the cell free binding assay format, both Rider et al. and Stoica et al. have reported similar results: CdCl_2_ prevents steroidal estrogens from binding to hERα. Based on our binding curve, the dissociation constant for cadmium was K_D_ = 6.1*10^−7^ M, which suggests strong but weaker interaction than reported by Stoica et al. (K_D_ = 5*10^−10^ M). The difference between the values could partly depend on the different experimental methods. While Stoica *et al.* utilized radioligand displacement assay, our platform is label-free and detects the binding event in real time. Other additional minor details, such as buffer composition, incubation times and reaction temperatures could further affect the result. Although the two K_D_ values differ, they both suggest that cadmium has marked affinity towards the hERα.

Although Rider et al. also observed interaction between cadmium and hERα in their cell free assay, they could not calculate a dissociation constant, because the data did not fit the one-site competitive model. In fact, also Stoica and coworkers noted that the binding of cadmium to hERα displayed non-competitive features. In agreement with these observations, we also observe that the shape of our data fits poorly to the binding curve (Figure 2A). The RIfS binding assay assumes competitive binding of ligands to the same ligand binding cavity following the mass equation [R]+[L]↔[RL]. Therefore, our data could reflect non-competitive binding mode in agreement with the earlier reports [8], [27]. The non-competitive binding mode, and subsequent difficulty to fit the data into a one-site model, could further explain the difference in the K_D_ value in our work compared to that of Stoica et al.

We next determined the effect of cadmium on the conformation of hERα. We incubated hERα with vehicle (buffer without ligand or CdCl_2_ but with corresponding amount of solvent), CdCl_2_, E_2_, the antiestrogen 4-OHT, or combinations of CdCl_2_ with E_2_ or 4-OHT. As expected, E_2_ treated hERα bound to the α/β I coated surface, indicating an agonist conformation, while vehicle and 4-OHT treated hERα did not (Figure 2B). To our surprise, CdCl_2_ treated hERα did not bind to the surface. Moreover, when hERα was co-incubated with E_2_ and CdCl_2_, it no longer bound to the surface (Figure 2B), indicating that cadmium destroys E_2_ induced agonist conformation of ERα.

In summary, these results suggest that cadmium binds to hERα with high affinity in a non-competitive manner without inducing an agonist conformation, and that cadmium disrupts the agonist conformation induced by E_2_. Based on these observations, it is more likely that cadmium would trigger antiestrogenic effects *in vivo* than estrogen agonist effects. Indeed, some reports in the literature support this hypothesis. Silva *et al.* did not observe effects on estrogen-dependent breast cancer cell proliferation with cadmium alone, but in the presence of E_2_ cadmium inhibited the proliferation [22]. Similar results were reported in yeast hERα transactivation assays [21], [22]. Furthermore, three independent *in vivo* studies suggest that cadmium does not promote uterine growth in rodents, suggesting lack of estrogen activity [23], [25], [34].

### Binding of cadmium to hERα with protected cysteine residues

Although the above results gain some support from the literature, we do realize that they contradict several other publications reporting estrogen-like effects for cadmium [6], [7], [8], [13], [14], [15], [16], [17], [18], [23], [24]. We were concerned that the high concentration of preservatives present in the commercial ERα preparation might have interfered with our assay. According to our calculations, the 537 nM hERα solution used in our assay contained a total concentration of 343 µM of ethylenediaminetetraacetic acid and dithiothreitol - both well known to complex Cd^2+^. Hence, we wanted to repeat the assays without preservatives. However, native ERα quickly destabilizes in the absence of additives due to unprotected cysteine residues. In order to solve this problem, we chose to use an hERα preparation consisting of the LBD (amino acids 301–553) with carboxymethylated cysteine residues [9]. This receptor variant is expected to maintain its normal ligand binding properties, as none of the LBD cysteine residues are in direct contact with the ligand [9]. In fact, we have previously utilized this receptor in the RIfS binding assay without problems [29].

**Figure 3:**
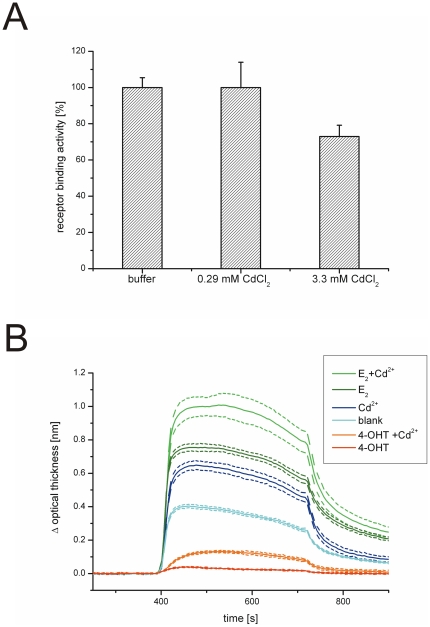
Effect of cadmium on CM-hERαLBD. (A) The binding of cadmium to CM-hERαLBD was tested by incubating different concentrations of CdCl_2_ (2.9 nM–285.7 µM) with a constant concentration of the receptor (208.3 nM). The data could not be fitted to a binding curve due to the absence of effects on the optical thickness. (B) To determine the conformation, CM-hERα-LBD was incubated with vehicle, CdCl_2_, E_2_, 4-OHT, CdCl_2_+E_2_, or CdCl_2_+4-OHT and guided to the α/β I coated sensor surface. The average binding curves (recorded for 900 sec) of three independent assays is shown with the standard deviation depicted as dotted lines. CdCl_2_ promoted the formation of the agonist conformation of the LBD alone as well as in combination with E_2_ and 4-OHT.

**Figure 4:**
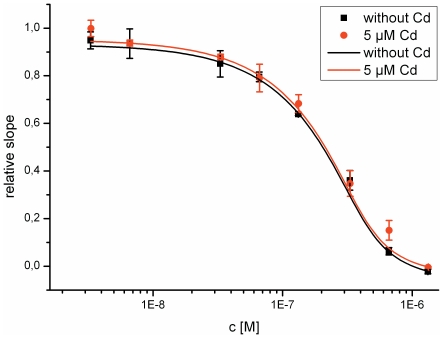
Effect of cadmium on E_2_ interaction with the CM-hERαLBD. The binding of E_2_ to CM-hERαLBD in the presence and absence of CdCl_2_ was studied by incubating different concentrations of E_2_ (ranging from 3.3 nM to 330.4 nM) with a constant concentration (208.3 nM) of the receptor in the absence (black curve) or presence (red curve) of 5 µM CdCl_2_. E_2_ dose dependently reduces optical thickness, and the presence of cadmium in the incubation solution did not alter the results.

We repeated the ligand binding and conformational assays using CM-hERαLBD instead of full length hERα. Unexpectedly, cadmium did not affect the binding of CM-hERαLBD to the sensor surface (Figure 3A). In the case of full length hERα, 300 µM CdCl_2_ completely blocked the receptor from binding to the surface (Figure 2A), but in the case of the CM-hERαLBD, the same concentration had no impact on the receptor, and with a 10-fold higher concentration only a minor reduction in the optical thickness was observed (Figure 3A). To control that the batch of CM-hERαLBD used in the assay functioned as it should, we measured the binding affinity of E_2_ to it. The obtained binding curve displayed a good fit (Figure 4, black curve) and the calculated K_D_ value of 2.0*10^−10^ M is in good agreement with reported values for full length, unprotected hERα [8], demonstrating that the batch of CM-hERαLBD at hand behaves like the native receptor. To further test the effect of cadmium on this receptor, we repeated the binding assay with E_2_ in the presence of cadmium. The E_2_ binding curves were essentially identical in the presence and absence of CdCl_2_, indicating that cadmium does not affect the binding of E_2_ to the CM-hERαLBD (Figure 4). These observations suggest that the LBD cysteine residues may have a central role in the binding of cadmium to hERα.

In addition to the protected cysteine residues, the CM-hERαLBD differs from the full length ERα in one more important aspect: it lacks the domains outside the LBD. The lack of the N-terminal A/B and central DNA binding domains could have an impact on the behavior of the receptor in our assay. Although both receptors variants we use behave normally in response to E_2_, the possibility remains that unorthodox ligands such as cadmium have a binding mode so distinct, that multiple domains are involved. The impact of cadmium on the DNA binding domain has been addressed before. Exchanging the zinc finger Zn^2+^ ions with Cd^2+^ has no functional consequence in terms of ERα DNA binding and transcriptional activity [35], [36]. We find it therefore unlikely that the presence or absence of this domain in our assay causes the dramatic change in the affinity of the receptor towards cadmium. The N-terminal A/B domain harbors the ligand independent activation function 1 (AF-1) that contributes to ER activity. Upon ligand binding, AF-1 and AF-2 can act together to synergistically boost ERα activity [37], and on the other hand, growth factors can activate ER in the absence of ligands through phosphorylation of serine residues in the AF-1 [38]. The synergistic activities of AF-1 and AF-2 involve co-regulator proteins that bridge these domains together, and the ligand independent activation through AF-1 relies on kinase activity. Our experimental setup does not contain co-regulators or kinases, and therefore we think that it is unlikely that the absence of AF-1 in the CM-hERαLBD is the reason for the discrepancy between the full length and truncated receptor affinities towards cadmium. It is more likely that this difference is due to the availability of the cysteine tails in the LBD, which have been shown to be crucial for cadmium-hERα interaction by other too [8], [39].

We further tested the effect of cadmium on the carboxymethylated receptor in the conformation assay. As expected, E_2_ induced agonist conformation while vehicle and 4-OHT did not (Figure 3B). However, to our great surprise, CdCl_2_ had marked effects on the conformation of the CM-hERαLBD. The binding curves show that the presence of CdCl_2_ in the reaction mixtures promotes the formation of the agonist conformation of the receptor in all environments: alone, in the presence of an agonist (E_2_), and in the presence of an antagonist (4-OHT) (Figure 3B). In summary, these observations suggest that cadmium does not bind to CM-hERαLBD in a manner that would prevent binding of E_2_ (*i.e.* not to the same ligand binding pocket), but cadmium does affect the conformation of the receptor. It seems as if cadmium, working outside the ligand binding pocket in the ERαLBD, can pull the receptor towards an active conformation. This would predict estrogen-like activities for cadmium in biological systems. Indeed, several studies have suggested that cadmium promotes estrogen signaling in cell culture and in experimental animals [6], [7], [8], [13], [14], [15], [16], [17], [18], [23], [24].

In conclusion, carboxymethylation of the cysteine residues in the LBD of hERα did not change the behavior of the receptor towards E_2_, but dramatically altered the behavior towards CdCl_2_. It is unlikely that these differences depend on the additives present in the commercial full length hERα, or domains outside the LBD. Instead, the results suggest fundamental differences in the binding mechanism between E_2_ and cadmium. Cadmium can coordinate with several amino acid tails, and a model has previously been proposed where cadmium interacts with cys381, cys447, glu523, his524 and asp538 in the hERα LBD [39]. The fact the mere carboxymethylation of the cysteine residues had such a great effect in our assays further emphasizes the role of these residues.

### A working hypothesis

The data presented here suggests that cadmium may interact with hERα in different manners depending on the state of the cysteine residues in the LBD of the receptor. The two models, one suggesting antagonistic and the other agonistic activity, both gain support from the literature. The burning question now is, whether both models could function in biological systems. In other words, can the state of the cysteine residues in the ERα LBD be affected in living cells?

The properties of many proteins are modified by post-translational modifications, where amino acid tails are for instance phosphorylated, sumoylated, or acetylated [40]. Various post-translational modifications have been reported to affect ERα, and although most of them are centered around the variable A/B domain in the N-terminus of the protein [41], the cys447 in the LBD is a target of palmitoylation [42]. Palmitoylation is a mechanism to associate proteins to cell membranes. Interestingly, cadmium has been shown to activate markers of membrane associated estrogen signaling in breast cancer cell lines *in vitro* as well as in rodents *in vivo* [14], [15], [24], [34]. The cysteine residues could also be modified in redox processes, a phenomenon that has so far been studied only in the DNA binding domain of ERα [43].

**Figure 5:**
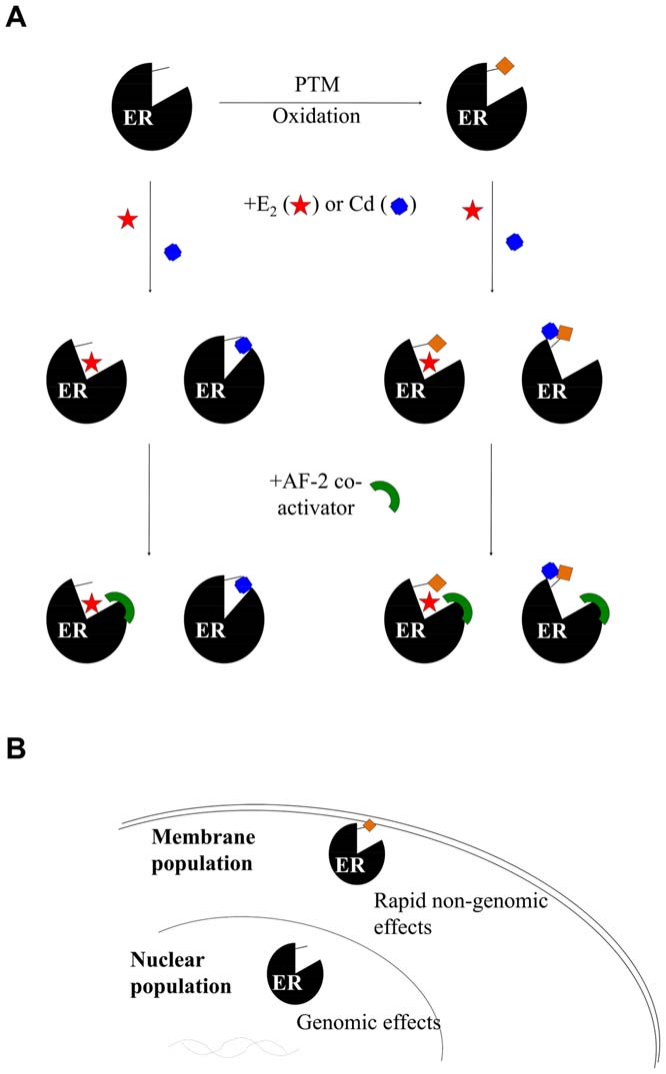
A working hypothesis. (A) Different cellular pools of ERα might have different reactivity towards cadmium depending on the cysteine residues (marked with a stick). The cysteine residues may become unavailable for interaction with cadmium for instance by post-translational modifications (PTM) or oxidation. When a total population of ERα is exposed to cadmium, the metal binds close the ligand binding pocket of unmodified ERα and prevents the formation of active conformation recognized by AF-2 interacting co-activators, but in the case of modified ERα (the diamond indicates modification of the cysteine residues) cadmium binds outside the ligand binding pocket and pulls the receptor towards the active conformation that allows interaction with co-activators. (B) In living cells, membrane-associated ERα and nuclear unmodified ERα could be examples of receptor pools with different reactivity towards cadmium.

Based on our results, we have formulated a working hypothesis where the effect of cadmium on estrogen signaling is a net result of different cellular pools of ERα responding to this heavy metal differently (Figure 5). The ERα cysteines could be affected for instance by oxidative stress (that cadmium can cause) or post-translational modifications, creating pools of ERα with a distinct reactivity towards cadmium (Figure 5A). This could lead to a hypothetical scenario where, for instance, palmitoylated ERα at the membranes is stimulated by cadmium whilst nuclear, unmodified is not (Figure 5B). Interestingly, support for this hypothetical model comes from two independent recent reports that have studied the effects of cadmium on both rapid, membrane associated estrogen signaling as well as on the nuclear estrogen signaling. Using human breast cancer cells, Zang et al. showed that cadmium promotes rapid ERK1/2 phosphorylation, which is a typical membrane associated estrogen effect, while not having an effect on the transcription of estrogen target genes pS2 and PgR [19]. Essentially the same pattern of activity was observed *in vivo* in mice by Ali and coworkers: they treated estrogen reporter mice with CdCl_2_ and observed phosphorylation of ERK1/2 in liver but no effects on transcriptional activity of the nuclear ERα [26]. It is worth noting that under suitable conditions extranuclear kinase activation can lead to transactivation of nuclear ERα [44]. The possible differing responsiveness of distinct cellular ERα pools to cadmium, combined with the inherent connection between the extranuclear and nuclear estrogen signaling, could help to explain some of the contradicting results on the estrogenicity of cadmium.

Future studies should vigorously test our working hypothesis by examining the effect of post-translational modifications and oxidative stress on the responsiveness of ERα towards cadmium in the nuclear and extranuclear parts of the estrogen signaling pathway.

## Supporting Information

Figure S1 **Surface chemistry reaction scheme.** Two differently functionalized transducers were used. Both were prepared in the same way for the first two steps: 1) Coupling of 3-glycidyloxypropyl-trimethoxysilane to the activated surface (in acetone for 1 h at room temperature) followed by 2) coupling of DAPEG (in dichloromethane for 18 h at 70°C). The transducers were then either 3) covalently modified with E_1_17-CMO (in DMF for 6 h at room temperature) using diisopropylcarbodiimide activation or 4) covalently modified with biotin (in DMF for 1 h at room temperature). The biotin-modified transducers were then put into the RIfS setup and rinsed with 5) Streptavin (1 mg/mL for 250 s at room temperature) and subsequently rinsed with 6) biotinylated α/β I peptide solution (1 mg/mL for 250 s at room temperature). The last two steps were also monitored online using RIfS.
(TIF)

Figure S2 **Ligand binding assay scheme.** A constant amount of hERa (548 nM for hERα and 208.3 nM for CM-hERαLBD) is incubated with different concentrations of estrogenic ligands (top row). With increasing amount of estrogenic ligand the binding of the receptor to the transducer chip is inhibited, resulting in a smaller increase in optical thickness and smaller relative slope. By plotting the relative slope values as a function of ligand concentration, and applying a non-linear fit (described in detail in the materials and methods section), the affinity of the ligand towards the receptor can be calculated.
(TIF)

Figure S3 **Conformation assay scheme.** A constant amount of hERa (3.3 µM) is incubated with either E_2_ (33 µM) or 4-OHT (24 µM) in the presence or absence of CdCl_2_ (3.3 mM). After the incubation phase of 1 h at 4°C, helix 12 of the receptor LBD is either in a typically agonistic conformation (symbolized by the cylinder in the green receptor, right) or in an antagonistic conformation (symbolized by the cylinder in the red receptor, left). When rinsing these mixtures over the α/β I modified transducer chip and monitoring the optical thickness as a function of time, the different conformations result in the different binding curves (higher green curve for agonists and lower red curve for antagonist) in RIfS.
(TIF)
