# Supplementary figures and images for: Biosensors Paving the Way to Understanding the Interaction between Cadmium and the Estrogen Receptor Alpha
Source: PLoS One. 2011 Aug 2;6(8):e23048. doi: 10.1371/journal.pone.0023048 (PMC3149063; doi:10.1371/journal.pone.0023048)

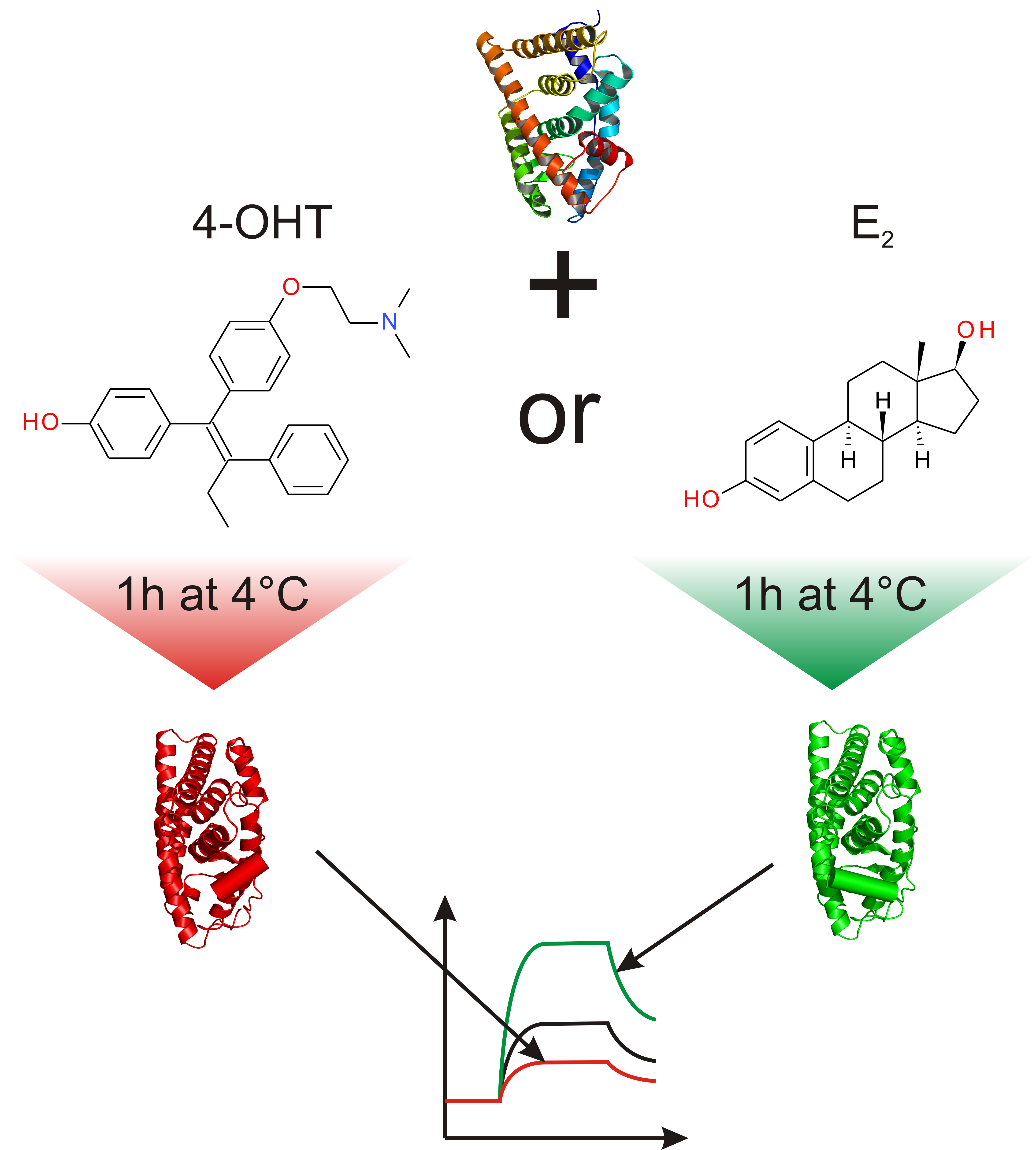

Supplement: Figure S3 — Conformation assay scheme. A constant amount of hERa (3.3 µM) is incubated with either E2 (33 µM) or 4-OHT (24 µM) in the presence or absence of CdCl2 (3.3 mM). After the incubation phase of 1 h at 4°C, helix 12 of the receptor LBD is either in a typically agonistic conformation (symbolized by the cylinder in the green receptor, right) or in an antagonistic conformation (symbolized by the cylinder in the red receptor, left). When rinsing these mixtures over the α/β I modified transducer chip and monitoring the optical thickness as a function of time, the different conformations result in the different binding curves (higher green curve for agonists and lower red curve for antagonist) in RIfS. (TIF) [file pone.0023048.s003.tif]
